# Nuclear Medicine Imaging in Neuroblastoma: Current Status and New Developments

**DOI:** 10.3390/jpm11040270

**Published:** 2021-04-04

**Authors:** Atia Samim, Godelieve A.M. Tytgat, Gitta Bleeker, Sylvia T.M. Wenker, Kristell L.S. Chatalic, Alex J. Poot, Nelleke Tolboom, Max M. van Noesel, Marnix G.E.H. Lam, Bart de Keizer

**Affiliations:** 1 Princess Maxima Center for Pediatric Oncology, Heidelberglaan 25, 3584 CS Utrecht, The Netherlands; a.samim-4@prinsesmaximacentrum.nl (A.S.); g.a.m.tytgat@prinsesmaximacentrum.nl (G.A.M.T.); S.T.M.Wenker-3@prinsesmaximacentrum.nl (S.T.M.W.); k.l.s.chatalic@umcutrecht.nl (K.L.S.C.); a.j.poot@umcutrecht.nl (A.J.P.); n.tolboom@umcutrecht.nl (N.T.); m.m.vannoesel@prinsesmaximacentrum.nl (M.M.v.N.); 2 Department of Radiology and Nuclear Medicine, University Medical Center Utrecht/Wilhelmina Children’s Hospital, Heidelberglaan 100, 3584 CX Utrecht, The Netherlands; m.lam@umcutrecht.nl; 3 Department of Radiology and Nuclear Medicine, Northwest Clinics, Wilhelminalaan 12, 1815 JD Alkmaar, The Netherlands; gbleeker78@gmail.com

**Keywords:** neuroblastoma, nuclear medicine, radionuclide imaging, [^123^I]mIBG, [^124^I]mIBG, [^18^F]mFBG, [^18^F]FDG, [^68^Ga]Ga-DOTA peptides, [^18^F]F-DOPA, [^11^C]mHED

## Abstract

Neuroblastoma is the most common extracranial solid malignancy in children. At diagnosis, approximately 50% of patients present with metastatic disease. These patients are at high risk for refractory or recurrent disease, which conveys a very poor prognosis. During the past decades, nuclear medicine has been essential for the staging and response assessment of neuroblastoma. Currently, the standard nuclear imaging technique is *meta*-[^123^I]iodobenzylguanidine ([^123^I]mIBG) whole-body scintigraphy, usually combined with single-photon emission computed tomography with computed tomography (SPECT-CT). Nevertheless, 10% of neuroblastomas are mIBG non-avid and [^123^I]mIBG imaging has relatively low spatial resolution, resulting in limited sensitivity for smaller lesions. More accurate methods to assess full disease extent are needed in order to optimize treatment strategies. Advances in nuclear medicine have led to the introduction of radiotracers compatible for positron emission tomography (PET) imaging in neuroblastoma, such as [^124^I]mIBG, [^18^F]mFBG, [^18^F]FDG, [^68^Ga]Ga-DOTA peptides, [^18^F]F-DOPA, and [^11^C]mHED. PET has multiple advantages over SPECT, including a superior resolution and whole-body tomographic range. This article reviews the use, characteristics, diagnostic accuracy, advantages, and limitations of current and new tracers for nuclear medicine imaging in neuroblastoma.

## 1. Introduction

### 1.1. Neuroblastoma

Neuroblastoma is the most common extracranial solid malignancy in children and more than 95% of patients are younger than 10 years of age. It is responsible for approximately 15% of all cancer deaths in children [1]. As an embryonic tumor derived from the sympathoadrenal lineage of the neural crest, neuroblastoma is classified as a neuroendocrine tumor. It can arise anywhere along the sympathetic trunk or adrenal medulla but most primary tumors are found in the abdomen (70%) [2]. About 50% of patients present with distant metastasis, which most commonly involves bone marrow, bone, and lymph nodes and less frequently involves the liver and skin [2,3].

Tumor stage is defined by the presurgical International Neuroblastoma Risk Group Staging System (INRGSS) [4] and the postsurgical International Neuroblastoma Staging System (INSS) [5]. Stage is the most important prognostic factor [6,7]. Other risk factors include age ≥ 18 months and unfavorable histopathological and/or biological features of the tumor [8].

The clinical course of disease is highly variable and ranges from spontaneous tumor regression to aggressive disease with fatal tumor progression. Despite intensive multimodality treatment, long-term survival of patients with high-risk neuroblastoma, comprising more than half of patients, is approximately 40% [9,10] and less than 20% in patients with refractory or relapsed disease [11,12].

### 1.2. Diagnostic Imaging

Radiological and nuclear medicine imaging are essential for the staging of neuroblastoma at diagnosis, along with the monitoring of treatment response and surveillance of any recurrent disease during follow-up. Radiological imaging of the chest, abdomen, and pelvis is required for morphological characterization of the primary tumor and to assess locoregional disease extent, such as soft tissue metastases and image-defined risk factors (IDRFs) representing the tumor’s relationship to adjacent vital structures [13]. Magnetic resonance imaging (MRI) is generally preferred over contrast-enhanced computed tomography (CT) due to its lack of radiation, higher soft tissue contrast resolution, and superior visualization of intraspinal extension [14].

For the detection of metastases in neuroblastoma, *meta*-[^123^I]iodobenzylguanidine ([^123^I]mIBG) planar whole-body scintigraphy is the current standard nuclear imaging technique. The addition of single-photon emission computed tomography with computed tomography (SPECT-CT) to planar scintigraphy has become standard in gamma camera imaging. SPECT-CT increases the certainty of interpretation and anatomical localization of small focal uptake [15,16,17].

### 1.3. Advances in Nuclear Imaging

With metastatic disease being an important prognostic factor, more accurate diagnostic methods for the assessment of disease extent are needed in order to optimize treatment strategies. Neuroblastomas express various molecular targets for nuclear medicine imaging. Several new tracers have been introduced that are compatible with positron emission tomography (PET) imaging in neuroblastoma, such as [^124^I]mIBG, [^18^F]mFBG, [^18^F]FDG, [^68^Ga]Ga-DOTA peptides, [^18^F]F-DOPA, and [^11^C]mHED.

PET imaging has multiple advantages over SPECT imaging [18,19]. First, PET has a higher spatial resolution and tumor-to-background contrast, enabling better detection of smaller lesions and better delineation of lesions. Second, PET is more suitable for quantification of tracer uptake, measured either as the standardized uptake value (SUV) or a percentage of the injected dose per unit volume. Third, PET enables whole-body tomographic reconstruction as opposed to the limited (±40 cm axial) field-of-view of SPECT. Fourth, PET acquisition is much faster (±15 min) compared to SPECT and scintigraphy (±90 min) [18,20]. This advantage is especially important for pediatric applications because a shorter scan time reduces the number and length of sedations and motion artifacts. Lastly, most PET-radioisotopes allow for same-day tracer administration and image acquisition.

This comprehensive review discusses the use, characteristics, diagnostic accuracy, and (dis)advantages of current and new tracers in the management of neuroblastoma.

## 2. [^123^I]mIBG

### 2.1. Uptake and Biodistribution

[^123^I]mIBG is *meta*-iodobenzylguanidine (mIBG), a norepinephrine analogue, labeled with gamma-emitting iodine-123 (^123^I). mIBG is taken up by the sympathicomedullary tissue via the norepinephrine transporter (NET), referred to as the uptake-1 system, and stored in neurosecretory granules [21]. As immature neuroblastoma cells contain few storage granules, mIBG is predominantly stored in the cytoplasm [22]. Physiological activity is seen in the liver, salivary glands, thyroid (if not blocked), nasal mucosa, gallbladder, myocardium, brown adipose tissue, adrenals, and urinary system (including the bladder). Occasionally, low diffuse activity is present in the colon, lungs, lacrimal glands, and spleen [20]. mIBG is not metabolized after administration and is almost entirely excreted by the kidneys via glomerular filtration. About 50% is excreted within the first 24 h after administration and 90% within 4 days [21].

### 2.2. Indication

For over 30 years, radiolabeled mIBG with either iodine-131 (^131^I) or ^123^I has been used as a tumor-specific agent for the imaging (and therapy) of neural crest tumors, such as neuroblastoma (including ganglioneuroblastoma), pheochromocytoma/paraganglioma, medullary thyroid carcinoma, and carcinoids [23]. The majority (90%) of neuroblastomas overexpress NET and actively accumulate mIBG [24].

For diagnostic imaging, [^123^I]mIBG is preferred over [^131^I]mIBG because [^123^I]mIBG provides better image quality at a lower radiation dose. [^123^I]mIBG has more favorable dosimetric properties compared to [^131^I]mIBG, including shorter half-life (13 h vs. 8 days), lower energy gamma emission (159 keV vs. 364 keV), and a lack of beta emission [23]. Therefore, [^123^I]mIBG scintigraphy is the first-line nuclear imaging used for the initial staging of neuroblastoma, monitoring of response, surveillance for recurrent disease during follow-up, and selection of eligible patients for [^131^I]mIBG radionuclide therapy [18,25].

### 2.3. Preparation

Antihypertensive agents, calcium channel blockers, sympathomimetics, central nervous system stimulants, tricyclic antidepressants, antipsychotics, antihistamines, and opioid analgesics are known to interfere with mIBG uptake. It is important to avoid these drugs (at least four times the biologic half-life) prior to [^123^I]mIBG imaging. Furthermore, thyroid-blocking medication is necessary to prevent the uptake and irradiation of the thyroid gland due to the presence of small amounts of free radioactive iodine [18].

Slow intravenous administration of [^123^I]mIBG over 1–5 min is recommended to avoid rare adverse reactions such as hypertension, tachycardia, nausea, and pallor. Planar whole-body scintigraphy, preferably with additional SPECT-CT, is performed 20–24 h after tracer administration. For all tracers that are renally excreted, voiding prior to imaging is important to limit radiation exposure to the bladder and enable adequate evaluation of the pelvis. Due to the long acquisition times (±90 min), sedation or general anesthesia is often necessary in young children [18].

### 2.4. Image Interpretation

Tracer uptake beyond the normal physiological distribution, for instance, any osteomedullary uptake, should be taken into consideration as pathological uptake [20]. One unequivocal mIBG-positive lesion at a distant site is sufficient to define metastatic disease, whereas one equivocal lesion requires confirmation by another imaging modality and/or biopsy [13]. Correlation of [^123^I]mIBG imaging with radiological imaging is important, as combined analysis has shown to increase diagnostic accuracy for lesion detection [26].

### 2.5. Prognostic Scoring Systems

**Figure 1:**
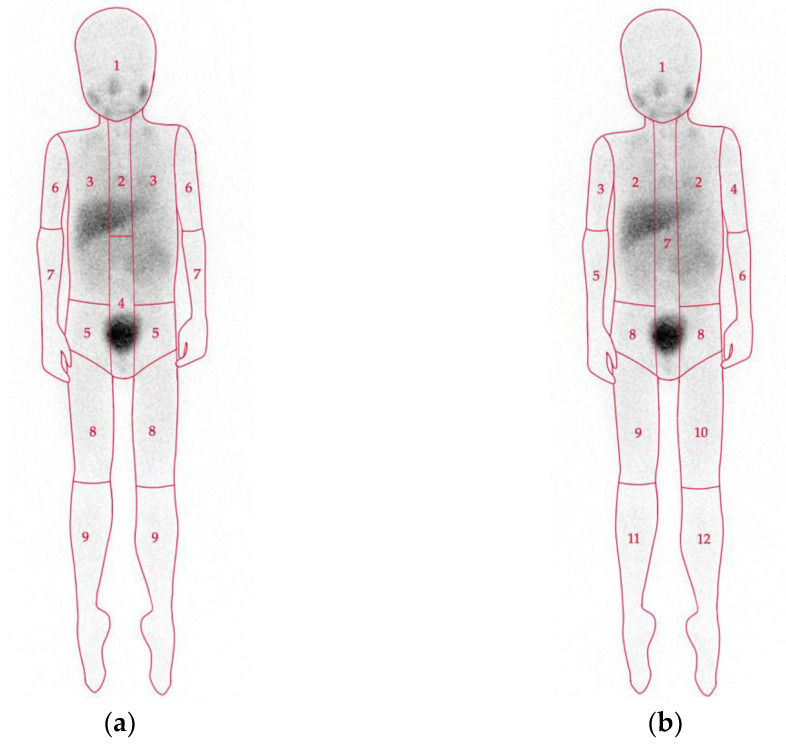
Scoring systems for mIBG scintigraphy. The Curie score (**a**) consists of 9 osteomedullary segments and a 10th soft tissue segment. Each segment is graded as: 0, no involvement; 1, one distinct lesion; 2, two or more distinct lesions; or 3, >50% involvement [27]. The International Society of Pediatric Oncology Europe Neuroblastoma Group (SIOPEN) score (**b**) consists of 12 osteomedullary segments. Each segment is graded as: 0, no involvement; 1, one distinct lesion; 2, two distinct lesions; 3, three distinct lesions; 4, four or more distinct lesions or <50% involvement; 5, >50% involvement; or 6, >95% diffuse involvement [28].

There are two widely used semiquantitative scoring systems that provide a standardized evaluation of the involvement of body segments on mIBG scintigraphy (Figure 1): The Curie score [27] and the International Society of Pediatric Oncology Europe Neuroblastoma (SIOPEN) score [28]. Large international clinical trials have shown that both scoring systems are equally reliable as prognostic indicators with respect to outcome (overall and event-free survival) [9,28,29]. Persisting metastatic disease at the end of induction chemotherapy is of great prognostic significance. Postinduction scores (Curie > 2 and SIOPEN > 3) can identify patients with a very poor outcome who require alternative treatment strategies [9,30,31].

### 2.6. Diagnostic Accuracy

[^123^I]mIBG imaging (scintigraphy and SPECT-CT) has high sensitivity and specificity (both around 90%) for neuroblastoma [24,32]. False-negative results can be a result of pharmacological interference.

However, without pharmacological interference, approximately 10% of neuroblastomas fail to accumulate mIBG [24,33,34]. This is the result of either insufficient NET expression or a modified mIBG uptake mechanism [35] of which the exact determinants are unclear. One of the possible explanations is tumor cell dedifferentiation, which has been supported to some extent by available studies [33,34,35,36,37]. However, this has not been confirmed. Patients with mIBG-negative disease are known to have superior outcomes compared to patients with mIBG-positive disease [30,36], and low mIBG uptake has been associated with tumors with low proliferative activity [33] Mixed patterns of both mIBG-positive and mIBG-negative disease may be present within the same patient or tumor [34]. Moreover, cases have been described of initially mIBG-positive tumors that were mIBG-negative at relapse [38,39] and vice versa [40]. Loss of mIBG uptake during treatment is of great concern because of the subsequent risk of missing any recurrent disease.

It is known that more differentiated neuroblastic tumors may likewise accumulate mIBG. Neuroblastoma lesions can mature to benign ganglioneuromas in response to chemotherapy and may remain mIBG-positive [41], although approximately 30% of ganglioneuromas are mIBG-negative [33,42]. In addition, rare benign uptake can occur in atelectasis, pneumonia, focal nodular hyperplasia, radiation injury of the liver, splenunculi, focal pyelonephritis, and vascular malformations [20]. Lastly, lesions in or close to areas of high physiological uptake may be missed. Physiological liver uptake is known to cause difficulty in identifying liver metastases.

### 2.7. Advantages and Limitations

Unlike MRI, [^123^I]mIBG imaging is unaffected by posttreatment changes, which contributes to the high specificity in the assessment of treatment response. Moreover, [^123^I]mIBG imaging can select eligible patients with mIBG-avid disease for [^131^I]mIBG therapy [25]. However, [^123^I]mIBG imaging has several limitations, such as limited spatial resolution, limited sensitivity for small lesions, prolonged acquisition sessions, limited tomographic field-of-view, need for pharmacological thyroid protection, and inconvenience of a 2-days schedule [18].

## 3. [^124^I]mIBG

### 3.1. Uptake and Biodistribution

mIBG imaging is also possible with PET radioisotope iodine-124 (^124^I). [^124^I]mIBG has a similar uptake mechanism and biodistribution to [^123^I]mIBG.

### 3.2. Indication

Only few reports have described the use of [^124^I]mIBG imaging in neuroblastoma concerning preclinical studies [43,44] and experimental studies in a total of eleven patients with neuroblastoma [45,46,47]. These reports studied the role of [^124^I]mIBG PET-CT for diagnostic imaging, as well as for dosimetric purposes before [^131^I]mIBG therapy.

### 3.3. Preparation

[^124^I]mIBG PET-CT has a similar preparation to [^123^I]mIBG imaging. PET-CT can be performed 1 day after tracer administration but also at later timepoints because of the long physical half-life of ^124^I (4.2 days). However, no recommended dose or optimal timing for acquisition have yet been established.

### 3.4. Diagnostic Accuracy

Cistaro et al. (2015) was the first to report the use of [^124^I]mIBG PET-CT in two patients with metastatic neuroblastoma. [^124^I]mIBG PET-CT detected more osteomedullary lesions in comparison to mIBG scintigraphy [45]. The most important limitation of diagnostic [^124^I]mIBG imaging, especially in the pediatric population, is the high radiation exposure. The effective dose (mSv/MBq) of [^124^I]mIBG is estimated to be approximately 20-times higher than that of [^123^I]mIBG for a 70 kg adult, and even up to 30-times higher for children under the age of 5 years [44,48]. Beijst et al. (2017) suggested that the administered [^124^I]mIBG activity could be limited to 10-times lower while maintaining an acceptable quality [44]. This was recently demonstrated by Aboian et al. (2020) in eight patients with neuroblastoma. With a lower dose, [^124^I]mIBG PET-CT still detected significant more lesions as compared to [^123^I]mIBG scintigraphy and SPECT-CT [47]. However, the effective doses of [^124^I]mIBG were still much higher: 15.5 mSv for a 63 kg child and 19.1 mSv for a 23 kg child, as compared to those calculated for [^123^I]mIBG: 4.7 mSv and 5.8 mSv, respectively (EANM dosage card version 01.02.2014 [49]).

### 3.5. Advantages and Limitations

[^124^I]mIBG was first investigated for the purpose of dosimetry before [^131^I]mIBG therapy [48,50], for which the additional radiation dose is neglectable [45]. The whole-body tomographic capability of PET imaging and relatively long half-life of ^124^I, similar to ^131^I, could provide a more personalized adjustment of administered [^131^I]mIBG activity to normal organs and tumor tissue. The feasibility of using [^124^I]mIBG PET for personalized dosimetry estimation for [^131^I]mIBG therapy was demonstrated in a neuroblastoma patient by Huang et al. (2015) [46].

Apart from the high radiation exposure for diagnostic purposes, [^124^I]mIBG has several other limitations. The complex decay scheme of ^124^I, with a positron abundance of only 23%, results in a relatively poorer image quality, compared to fluorine-18 (^18^F) or gallium-68 (^68^Ga)-labeled radiotracers [51]. Moreover, cyclotron-produced [^124^I]mIBG is not widely available for clinical use, which is accompanied by high costs and lack of experience. There is still need for pharmacological thyroid protection and the inconvenience of a 2-days imaging schedule.

## 4. [^18^F]mFBG

### 4.1. Uptake and Biodistribution

*meta*-[^18^F]Fluorobenzylguanidine ([^18^F]mFBG) is the fluorinated analog of mIBG, labeled with PET radioisotope ^18^F. [^18^F]mFBG accumulates in cells via the same norepinephrine transporter uptake mechanism as [^123^I]mIBG, with a biodistribution similar to that of [^123^I]mIBG. However, the more hydrophilic [^18^F]mFBG has the advantage of much faster tissue uptake and renal clearance. One hour after administration, 45% of the administered activity is already excreted, and after 3–4 h, up to 95% [52,53]. A preclinical study by Zhang et al. (2014) reported that tumor uptake of [^18^F]mFBG was three-times higher than [^123^I]mIBG in vivo [52].

### 4.2. Indication

[^18^F]mFBG is a new experimental tracer developed for diagnostic PET imaging of NET-expressing tumors. The use of [^18^F]mFBG has only been reported in eleven human cases, five of which were patients with neuroblastoma [53,54].

### 4.3. Preparation

Due to limited experience, no recommended dose or optimal timing for acquisition have been established. Acquisition 1–2 h after [^18^F]mFBG administration has been proposed [53]. In contrast to [^123^I]mIBG there is no need for pharmacological thyroid protection due to the absence of radioactive iodine.

### 4.4. Diagnostic Accuracy

Pandit-Taskar et al. (2018) conducted the first human study on [^18^F]mFBG in five patients with neuroblastoma and five patients with pheochromocytoma/paraganglioma [53]. [^18^F]mFBG PET-CT detected all 63 lesions detected on [^123^I]mIBG imaging (scintigraphy and SPECT-CT) and 59 additional lesions.

### 4.5. Advantages and Limitations

**Figure 2:**
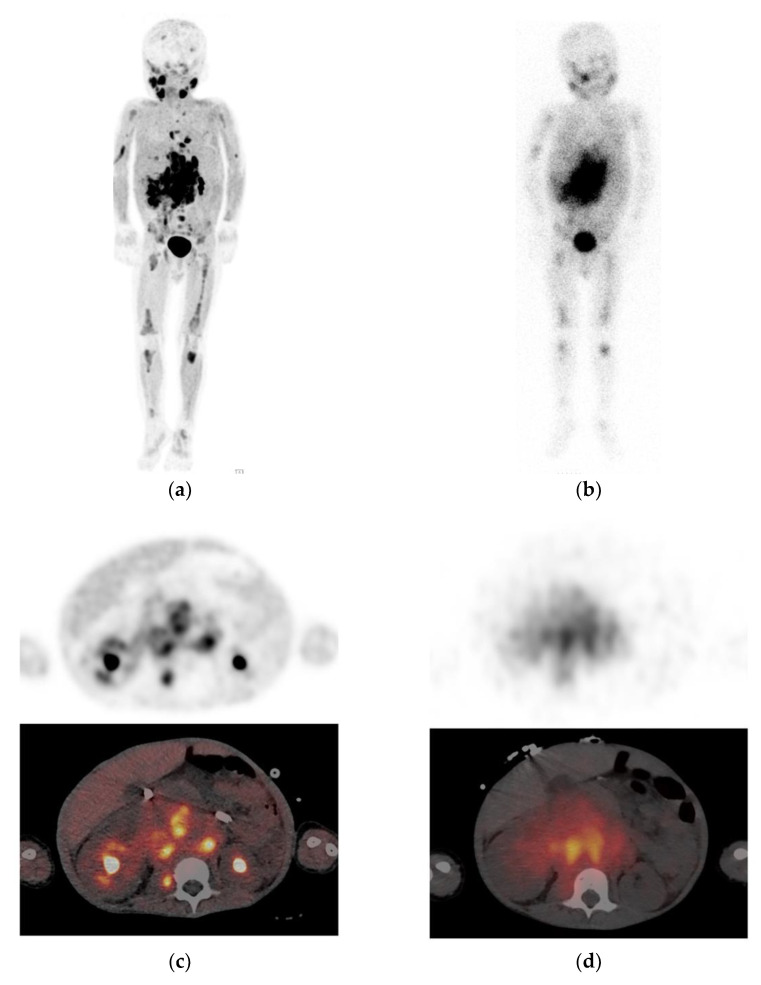
Example of a neuroblastoma patient with extensive metastasis who underwent [^123^I]mIBG and [^18^F]mFBG imaging within 1 day. These figures illustrate the superior image quality and tumor delineation of (**a**) [^18^F]mFBG PET maximum intensity projection and (**c**) axial PET-CT due to higher spatial resolution, higher tumor-to-background contrast, and improved counting statistics compared to (**b**) [^123^I]mIBG planar scintigraphy and (**d**) axial single-photon emission computed tomography with computed tomography (SPECT-CT).

Besides the general advantages of PET imaging compared to scintigraphy/SPECT (Figure 2), the radioisotope ^18^F has an ideal half-life of 110 min, which allows for 1-day imaging. [^18^F]mFBG PET-CT was found to be safe and feasible [53]. Despite the shorter physical half-life of ^18^F and biologic half-life of [^18^F]mFBG, radiation exposure is comparable to that of [^123^I]mIBG, because of the high-energy ^18^F emissions [52]. Disadvantages of cyclotron-produced [^18^F]mFBG are the limited availability and lack of experience.

## 5. [^18^F]FDG

### 5.1. Uptake and Biodistribution

[^18^F]Fluorodeoxyglucose ([^18^F]FDG) is a glucose analogue labeled with the PET radioisotope ^18^F. As a metabolic compound, [^18^F]FDG uptake reflects anatomical locations with high glucose metabolism. [^18^F]FDG is transported into the cell via passive glucose transporters and intracellularly phosphorylated by hexokinase into [^18^F]FDG-6-phosphate. Unlike glucose-6-phosphate, [^18^F]FDG-6-phosphate cannot be dephosphorylated or further metabolized in the glycolytic process and steadily accumulates in metabolically active cells [55]. 

Physiological activity is seen in the brain, salivary glands, Waldeyer’s ring, myocardium, thymus, brown adipose tissue, liver, spleen, and gastrointestinal tract. [^18^F]FDG is excreted by the kidneys. Therefore, the urinary system shows prominent activity. Physiological bone marrow activity in children is variable, but flat and symmetrical uptake is usually present in epiphyseal growth plates [56].

### 5.2. Indication

Malignant tissues strongly accumulate [^18^F]FDG because of an increased glucose metabolism rate [56]. [^18^F]FDG PET-CT is increasingly used in pediatric malignancies, including neuroblastoma. Several studies have confirmed that the majority of neuroblastoma lesions concentrate [^18^F]FDG [57,58,59,60,61,62]. [^18^F]FDG frequently accumulates in mIBG non-avid neuroblastoma due to a different uptake mechanism that is not dependent on NET expression [32,58], [^18^F]FDG is the only PET-tracer that has been recommended by the current guidelines for the following two indications [18]. First, as a replacement of [^123^I]mIBG in the evaluation of mIBG-negative or weakly mIBG-positive neuroblastoma, and second, as complementary modality when radiological imaging or clinical findings suggest more extensive disease than is revealed by [^123^I]mIBG imaging. [^18^F]FDG has taken over the role of technetium-99m (^99m^Tc) skeletal scintigraphy, which has long been the second-line imaging to assess bone metastases in mIBG-negative tumors or in the case of solitary equivocal mIBG bone uptake [63,64].

### 5.3. Preparation

Regarding interactions with [^18^F]FDG, there is only evidence for interaction with glucose. Fasting and discontinuing any intravenous glucose administration is recommended for at least 4 h prior to [^18^F]FDG administration [18]. Right before administration, blood glucose levels should be below 7 mmol/L. To prevent physiological [^18^F]FDG uptake by brown adipose tissue and peripheral muscles, it is important to let the patient rest in a warm environment. Some centers use additional premedication such as propranolol prior to [^18^F]FDG administration [65]. Acquisition is generally started 60 min postadministration.

### 5.4. Diagnostic Accuracy

Overall, most studies have reported that [^18^F]FDG PET cannot replace [^123^I]mIBG imaging in mIBG avid neuroblastoma due to lower sensitivity and specificity, especially for osteomedullary lesions, the most frequent site of metastases [57,58,60,61,66]. This is in contrast with the study of Kushner et al. (2001) in 51 high-risk neuroblastoma patients, who suggested that, in the absence of skull lesions and after primary tumor resection, [^18^F]FDG PET and bone marrow sampling would suffice for disease monitoring in high-risk patients [62]. There is evidence that [^18^F]FDG may be superior in localized neuroblastoma due to better depiction of locoregional soft tissue disease [57,66]. All studies conjointly support the value of the [^18^F]FDG PET in mIBG non-avid neuroblastoma and the complementary role in case of discrepant findings between mIBG imaging and radiological/clinical findings. Melzer et al. (2011) demonstrated the complementary role of [^18^F]FDG PET to [^123^I]mIBG imaging (scintigraphy and SPECT) in 19 patients with [^123^I]mIBG non-avid tumors or discrepant findings. [^18^F]FDG PET correctly identified 32 out of 34 discrepant lesions. In this setting, [^18^F]FDG PET was more sensitive (78% vs. 50%) and specific (92% vs. 75%) than [^123^I]mIBG imaging for the detection of neuroblastoma lesions [59].

It is important to realize that [^18^F]FDG-positive and [^123^I]mIBG-negative lesions (and vice versa) can coexist in the same patient [67,68,69]. When [^123^I]mIBG uptake is absent or lost during disease course, [^18^F]FDG may still show pathological uptake. Generally, poorly differentiated tumors tend to show high [^18^F]FDG uptake due to high glucose metabolism [38]. On the other hand, persisting posttherapy [^123^I]mIBG activity but normal [^18^F]FDG distribution is also possible [70,71]. This may indicate that the tumor has matured to a benign ganglioneuroma, which can be confirmed by biopsy [66].

By reflecting increased metabolism, [^18^F]FDG is not a tumor-specific agent. [^18^F]FDG can therefore accumulate in nonmalignant lesions, as often seen in infection/inflammation [56]. Misinterpretation can be minimized by correlation with radiological imaging and a complete clinical history. Nevertheless, false-positive results are relatively common. Increased bone marrow activity can be seen in physiological bone marrow hyperplasia, which can be the result of chemotherapy or granulocyte colony-stimulating factor treatment [56]. On [^18^F]FDG PET, this is generally seen as diffuse activity, but focal activity is also possible, which may mimic metastatic bone marrow disease [57,58,60]. Therefore, [^18^F]FDG PET imaging is less optimal for assessment of treatment response of osteomedullary lesions.

Also, not all neuroblastoma lesions show elevated [^18^F]FDG activity, causing false-negative results of lesions with slow metabolic activity or necrosis. Furthermore, [^18^F]FDG is associated with the impaired detection of small lesions in the bone marrow and skull because of the presence of (nearby) physiological activity [60,62,66].

### 5.5. Advantages and Limitations

A major advantage of [^18^F]FDG PET-CT is that it is widely available. Moreover, several studies have reported that more prominent [^18^F]FDG activity (SUV_max_) of primary tumor and metastases is significantly correlated with decreased survival [60,72,73,74,75]. Therefore, [^18^F]FDG uptake may assist in the identification of patients with poor prognosis.

## 6. [^68^Ga]Ga-DOTA Peptides

### 6.1. Uptake and Biodistribution

Gallium-68 (^68^Ga)-labeled DOTA-Tyr^3^-octreotate (DOTATATE), DOTA-Tyr^3^-octreotide (DOTATOC), and DOTA-Nal^3^-octreotide (DOTANOC) are somatostatin analogues that are compatible with PET imaging. The five subtypes of somatostatin receptors (SSTR1–SSTR5) are expressed by many cells, mainly of the central and peripheral nervous system, endocrine glands, and gastrointestinal tract [76].

High physiological activity of [^68^Ga]Ga-DOTA peptides is seen in the spleen, adrenal glands, liver, kidneys, urinary system, and pituitary, and moderate uptake in the salivary glands, thyroid, pancreas, and gastrointestinal tract. Uptake in the pancreas is often heterogenous and low-grade uptake, but prominent uptake may occur in the uncinate process. Within 4 h, all the administered activity is excreted, almost entirely by renal clearance [18,77,78,79].

[^68^Ga]Ga-DOTA peptides have different affinity profiles for SSTR subtypes, which may lead to small differences in biodistribution. Whereas the affinity profile of [^68^Ga]Ga-DOTATATE is limited to SSTR2, [^68^Ga]Ga-DOTATOC and [^68^Ga]Ga-DOTANOC have a wider receptor binding profile. [^68^Ga]Ga-DOTATOC has high affinity for SSTR2 and SSTR5 and [^68^Ga]Ga-DOTANOC for SSTR2, SSTR3, and SSTR5. Clinical choice of a particular [^68^Ga]Ga-DOTA peptide is often driven by local availability because diagnostic performance appears to be comparable [77,80,81].

### 6.2. Indication

In particular, SSTR2 is highly expressed by neuroendocrine tumors such as gastroenteropancreatic neuroendocrine tumors, pheochromocytoma/paragangliomas [76], and most neuroblastomas (60–90%) [37,82,83,84]. High SSTR2 expression is mainly seen in more differentiated neuroblastoma [37]. [^68^Ga]Ga-DOTA peptide imaging (and peptide receptor radionuclide therapy [PRRT]) is already well established in adult patients with SSTR-positive neuroendocrine tumors [77]. Despite increasing off-label use in mIBG non-avid neuroblastoma, there is still no established role for [^68^Ga]Ga-DOTA peptide imaging in current guidelines.

### 6.3. Preparation

Somatostatin analog therapy may interfere with tracer distribution. Although evidence is lacking, it is recommended to avoid all short- and long-acting somatostatin agents. Acquisition is typically started 60 (range: 45–90) minutes after tracer administration [18].

### 6.4. Diagnostic Accuracy

The limited studies in patients with neuroblastoma suggest a higher sensitivity for lesion detection of [^68^Ga]Ga-DOTA peptide imaging compared to [^123^I]mIBG imaging.

Kroiss et al. (2011) compared [^68^Ga]Ga-DOTATOC PET with [^123^I]mIBG imaging (scintigraphy and SPECT) in four patients with neuroblastoma. [^68^Ga]Ga-DOTATOC PET detected a total of 132 lesions, and [^123^I]mIBG imaging detected 100 lesions. It is unknown whether [^68^Ga]Ga-DOTATOC PET detected all lesions found on [^123^I]mIBG imaging. Of the 107 lesions identified on CT/MRI, [^68^Ga]Ga-DOTATOC PET detected 104 lesions (97.2%, *p* < 0.0001) and [^123^I]mIBG SPECT detected 97 lesions (90.7%, *p* < 0.09) [85].

Kong et al. (2016) compared [^68^Ga]Ga-DOTATATE PET-CT to mIBG imaging (pretreatment [^123^I]mIBG scintigraphy and SPECT-CT or posttreatment [^131^I]mIBG scintigraphy and SPECT) in eight patients with refractory neuroblastoma [86]. [^68^Ga]Ga-DOTATATE uptake and immunohistological SSTR2 staining were positive in all patients and tumor samples, respectively. Even the smallest sub-centimeter lesions showed high tumor-to-background contrast and high SUV values. In three patients, [^68^Ga]Ga-DOTATATE PET-CT identified additional disease compared to mIBG imaging. However, in two of these three patients, the time between paired scans was about 10 weeks. One patient underwent biopsy of a bone marrow lesion, which was histologically confirmed to be metastasis. Several tumor samples only showed mild SSTR2 staining but high uptake on [^68^Ga]Ga-DOTATATE imaging. The authors postulated that the samples may have been collected from a tissue area with a lower expression and density of SSTR2.

Gains et al. (2020) compared [^68^Ga]Ga-DOTATATE PET maximum intensity projection (MIP) images to [^123^I]mIBG planar scintigraphy in 42 high-risk neuroblastoma patients. [^68^Ga]Ga-DOTATATE was positive in all patients, whereas [^123^I]mIBG was positive in 40 patients. [^68^Ga]Ga-DOTATATE identified bone lesions in 97% (35/36) of the patients vs. 81% (29/36) for [^123^I]mIBG and identified soft-tissue lesions in 100% (33/33) vs. 88% (29/33) of patients, respectively. Seven patients had [^68^Ga]Ga-DOTATATE-positive bone lesions with negative [^123^I]mIBG scans, whereas only one patient had [^123^I]mIBG-positive bone lesions and a negative [^68^Ga]Ga-DOTATATE PET. SIOPEN scores for [^68^Ga]Ga-DOTATATE were significantly higher compared to [^123^I]mIBG. [^68^Ga]Ga-DOTATATE detected more (osteomedullary and soft tissue) lesions than [^123^I]mIBG in 31–52% of patients, whereas [^123^I]mIBG detected more lesions than [^68^Ga]Ga-DOTATATE in 5% of patients [87].

**Figure 3:**
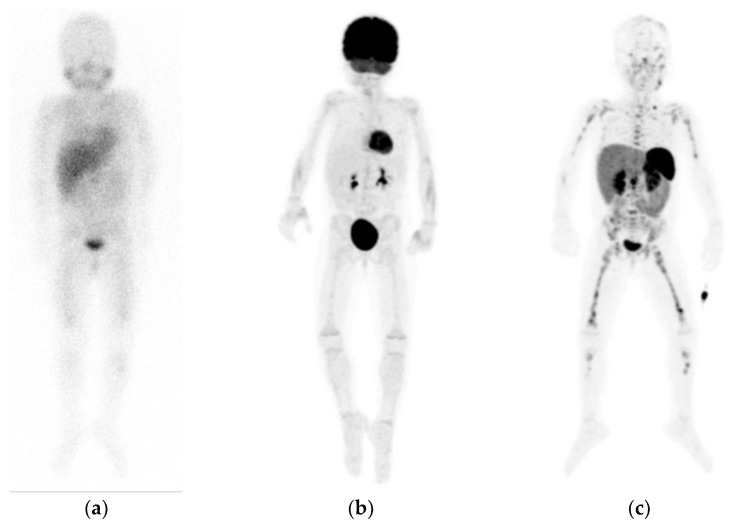
Example of a patient with neuroblastoma, showing diffuse pathological but faint osteomedullary uptake on planar [^123^I]mIBG scintigraphy (**a**), without increased uptake on [^18^F]FDG positron emission tomography (PET) maximum intensity projections (MIP) (**b**), but extensive pathological osteomedullary uptake on [^68^Ga]Ga-DOTATATE PET MIP (**c**). Note the different physiological distribution between tracers.

With completely negative or weakly positive [^123^I]mIBG imaging, extensive metastatic disease on [^68^Ga]Ga-DOTA peptide imaging may be present (Figure 3). This has been described by several cases of neuroblastoma patients [79,88,89]. On the other hand, [^68^Ga]Ga-DOTA peptide imaging may be negative in tumors with low SSTR expression, more likely seen in poorly differentiated tumors [90].

Physiological tracer distribution can potentially mask small-volume disease, for instance, when liver metastases show a similar degree of tracer accumulation to the normal liver. As SSTR uptake is not specific for tumoral pathologies, false-positive results can occur. Splenunculi or benign meningiomas may mimic focal metastatic disease. Uptake, usually low-grade, can be seen at sites of inflammation/infection, postradiotherapy changes, and osteoblastic activity (fractures, vertebral hemangioma, or epiphyseal growth plates) [78].

### 6.5. Advantages and Limitations

First, [^68^Ga]Ga-DOTA peptide imaging has shown to be safe and feasible in pediatric patients [18,79]. ^68^Ga is derived from generators and, therefore, more easily available and practical for centers that do not have cyclotrons. Its short half-life (68 min) allows for 1-day imaging and results in a lower radiation exposure than [^123^I]- and [^18^F]-labeled tracers [18,91].

Second, [^68^Ga]Ga-DOTA peptide uptake can have prognostic value. Low uptake has been shown to be an indicator of poor prognosis in adults with neuroendocrine tumors [92,93,94], but this has not been studied in neuroblastoma. Nevertheless, higher SSTR2 expression is correlated with higher [^68^Ga]Ga-DOTATATE uptake [94]. Several studies found that higher SSTR2 expression is strongly associated with non-high-risk and differentiated neuroblastoma, and lack of SSTR expression has been found to correlate with advanced disease and decreased survival [82,90,95,96,97], while others did not find any prognostic relation [37].

Third, confirmation of [^68^Ga]Ga-DOTA peptide uptake can be used to identify potential candidates for peptide receptor radionuclide therapy (PRRT) by replacing ^68^Ga with lutetium-177 (^177^Lu) [94]. The safety and feasibility of PRRT has been demonstrated in small groups of patients with refractory or relapsed neuroblastoma [86,98,99]. Gains et al. (2011) demonstrated that six out of eight patients with relapsed or refractory neuroblastoma showed sufficient [^68^Ga]Ga-DOTATATE uptake (higher than the liver) to be treated with experimental [^177^Lu]Lu-DOTATATE [98]. Recently, the same authors conducted a phase II trial, which was unfortunately terminated prematurely due to a lack of objective response and dose-limiting toxicity in one of the patients [100].

Last, along with the general advantages of PET imaging, [^68^Ga]Ga-DOTA peptides have practical advantages such as simple patient preparation and few pharmacological interactions.

## 7. [^18^F]F-DOPA

### 7.1. Uptake and Biodistribution

[^18^F]Fluoro-L-DOPA ([^18^F]F-DOPA) is L-DOPA (L-dihydroxyphenylalanine), the precursor of dopamine, norepinephrine, and epinephrine, labeled with PET radioisotope ^18^F. [^18^F]F-DOPA is a metabolic compound that reflects locations of increased catecholamine metabolism. [^18^F]F-DOPA is actively transported into cells via the large neutral amino acid transporter 1 (LAT1) system and subsequently converted by the enzyme L-amino acid decarboxylase (AADC) into [^18^F]fluorodopamine [101]. In physiological situations, dopamine is stored intravesicularly and converted into norepinephrine and epinephrine [102]. Uptake and retention of [^18^F]F-DOPA in neuroblastoma cells is dependent on increased intracellular transport and activity of AADC and less dependent on intravesicular storage [18,103].

Physiological activity is seen in the basal ganglia, pancreas, kidneys, adrenal glands, and epiphyseal growth plates in children [101,104]. Very intense activity is seen in the excretory tracts of the urinary system and biliary system [18]. Uptake in the pancreas and adrenal glands can occasionally be prominent, for instance, in the uncinate process of the pancreas. Mild diffuse uptake can be seen in the liver, myocardium, peripheral muscles, or gastrointestinal tract [77].

### 7.2. Indication

As a tumor-specific tracer, [^18^F]F-DOPA can be used for the imaging of neuroendocrine tumors with increased catecholamine metabolism, mainly seen in tumors of the sympathetic nervous system. The main indication is as alternative to [^68^Ga]Ga-DOTA peptide imaging [77].

[^18^F]F-DOPA may be a promising PET alternative to [^123^I]mIBG. Due to limited experience, [^18^F]F-DOPA has no evident role in the current guidelines for the evaluation of neuroblastoma. In some clinics [^18^F]F-DOPA is used as an off-label alternative tracer in mIBG-negative or weakly mIBG-positive neuroblastomas [18].

### 7.3. Preparation

Patients must fast for at least 4 h before tracer administration to avoid interaction with amino acids from food. Despite the lack of evidence, it is recommended to avoid AADC inhibitors such as carbidopa, catechol-*O*-methyl transferase inhibitors, and monoamine oxidase A inhibitors at least 48 h prior to tracer administration [18]. There is some evidence that carbidopa may actually reduce the physiological uptake of abdominal organs, but this effect is not fully elucidated [105]. Images are usually acquired 60–90 min postadministration [18].

### 7.4. Diagnostic Accuracy

The prospective pilot studies of Piccardo et al. (2012) and Lu et al. (2013) found that [^18^F]F-DOPA PET-CT detected a significantly higher number of neuroblastoma lesions compared to [^123^I]mIBG scintigraphy [103,106]. As SPECT-CT was not performed by all investigations, Piccardo et al. (2020) recently conducted another prospective study in 18 neuroblastoma patients comparing [^18^F]F-DOPA PET-CT with [^123^I]mIBG imaging (scintigraphy and SPECT-CT) using histological results or anatomical imaging (CT or MRI) as the reference standard [107]. [^18^F]F-DOPA PET-CT was more sensitive than [^123^I]mIBG SPECT-CT in detecting soft tissue lesions and small osteomedullary lesions, both at diagnosis and after chemotherapy. On a lesion-based analysis, the sensitivity at diagnosis for detecting soft tissue lesions was 86% for [^18^F]F-DOPA vs. 41% for [^123^I]mIBG, and for detecting osteomedullary lesions, 99% vs. 93%, respectively. After chemotherapy, the sensitivity for detecting soft tissue lesions was 77% [^18^F]F-DOPA vs. 28% % for [^123^I]mIBG, and for detecting osteomedullary lesions, 86% vs. 69%, respectively (all *p* < 0.001). It appears that even with a completely negative [^123^I]mIBG or [^18^F]FDG scan, [^18^F]F-DOPA PET-CT can still reveal persistent disease [103,107].

In addition, false-positive findings as a result of atypical physiological uptake have been reported, for instance, in the case of asymmetrical intense uptake in one of the adrenal glands or the presence of biliary duct stasis [104].

### 7.5. Advantages and Limitations

[^18^F]F-DOPA uptake seems to be of prognostic value. Piccardo and colleagues developed the [^18^F]F-DOPA whole-body metabolic burden (WBMB) score, calculated as the sum of bone metabolic burden (SUV_mean_ × SIOPEN score) of each bone-segment and the soft tissue metabolic burden (SUV_mean_ × volume) of each soft tissue lesion [108]. A higher disease burden (WBMB > 7.5) after induction was independently and significantly associated with disease progression and mortality [107,108]. In contrast, Liu et al. (2017) found that lower [^18^F]F-DOPA uptake (SUV_max_ and metabolic active primary tumor volume) is related to a poor prognosis, and the authors contemplated this may be due to dedifferentiation on a molecular level [72]. There is a limited availability of this new tracer and lack of experience in pediatric applications.

## 8. Other

### 8.1. [^11^C]mHED

Carbon-11 (^11^C)-labeled *meta*-hydroxyephedrine ([^11^C]mHED) is another norepinephrine analogue. The very hydrophilic [^11^C]mHED shows rapid accumulation (within minutes) in sympathicomedullary tissue via NET and high retention in neural crest tumors [109]. Because of fast blood clearance by the liver and kidney, an optimal imaging time of 30 min after tracer administration was proposed [110]. The only two studies on [^11^C]mHED imaging in neuroblastoma have demonstrated high diagnostic accuracy in the detection of neuroblastoma lesions. In six patients, Shulkin et al. (1996) showed that [^11^C]mHED PET identified all lesions found on [^123^I]mIBG scintigraphy, and SPECT and detected additional skull lesions in one patient [110]. After that Franzius et al. (2006) concluded that [^11^C]mHED may be slightly less sensitive than [^123^I]mIBG [111]. In one of six neuroblastoma patients [^11^C]mHED PET-CT missed one large abdominal relapse that was visible on [^123^I]mIBG imaging (scintigraphy and SPECT-CT). [^11^C]mHED detected all other lesions detected by [^123^I]mIBG imaging without detecting any additional lesions. Prominent physiological activity of [^11^C]mHED is seen in the liver, kidneys, and urinary system, often exceeding tumor uptake, which may impede detection of small metastases in these regions [110,111]. The short half-life of ^11^C (20 min) is a benefit because radiation exposure is considerably lower compared to [^123^I]mIBG [110]. However, the short half-life is also the largest limitation because of the requirement of an onsite cyclotron, a complicated radiochemical labeling procedure, and a rigid time schedule [111]. 

### 8.2. Other Benzylguanidine Analogues

In recent years, many other benzylguanidine analogues have been developed [112]. ^18^F-labeled fluoropropylbenzylguanidine (FPBG) PET-CT detected one extra (histologically confirmed) metastatic bone lesion compared to [^123^I]mIBG scintigraphy in a case report of a neuroblastoma patient [113]. Other analogues that have only been studied in neuroblastoma cell lines are parafluorobenzylguanidine (PFBG) [114], LMI1195 (N-[3-bromo-4-(3-F-fluoro-propoxy)-benzyl]-guanidine) [115], FPOIBG (4-fluoropropoxy-3-iodobenzylguanidine) [116], and *meta*-bromobenzylguanidine (mBBG) [117].

### 8.3. Potential Theranostics

[^123^I]/[^131^I]mIBG and [^68^Ga]/[^177^Lu]Ga-DOTA peptides are examples of a theranostic approach, which involves the use of the same molecular target for diagnostic imaging and radionuclide therapy. These theranostics are still under investigation in clinical trials. New potential theranostics for neuroblastoma have been studied in preclinical studies, such as iodine-125 (^125^I) labeled GPAID ((R)-(-)-5-[^125^I]iodo-3’-O-[2-(ε-guanidinohexanoyl)-2-phenylacetyl]-2’-deoxyuridine), a norepinephrine analogue that co-targets DNA of proliferating cells [118]; an antagonist of CXC chemokine receptor 4 (CXCR4), frequently overexpressed in various tumor types in the form of ^68^Ga-labeled pentixafor and ^177^Lu-labeled pentixather [119]; and zirconium-89 (^89^Zr)-labeled dinutuximab, a radiolabeled anti-GD2 immunotherapy [120].

## 9. Discussion

In this review, the use, characteristics, advantages and limitations of several new PET radiotracers for imaging of neuroblastoma are discussed (Table A1). At the moment, [^123^I]mIBG scintigraphy (with SPECT-CT) is the best established and first-line nuclear imaging technique in neuroblastoma patients. However, there is an increasing use of PET imaging in neuroblastoma with multiple advantages over SPECT, such as superior resolution, full body tomographic range, accurate quantification of tracer uptake and the convenience of shorter acquisition times and often allowing for one-day imaging.

Regarding the diagnostic accuracy of PET imaging for lesion detection in neuroblastoma, preliminary reports in small patient cohorts have shown overall promising results. [^124^I]mIBG, [^18^F]mFBG, [^68^Ga]Ga-DOTA peptides, and [^18^F]F-DOPA appear to be sensitive and specific tracers, possibly even more sensitive than [^123^I]mIBG imaging, especially for the detection of smaller lesions that are below the spatial resolution of SPECT. Only [^18^F]FDG PET imaging seems less sensitive and specific than [^123^I]mIBG imaging. Nevertheless, the difficulty of calculating sensitivity and specificity of PET imaging in neuroblastoma is that there is no real gold standard by which to measure its accuracy [78]. As histological confirmation of pathological uptake is often not feasible due to ethical and practical reasons, most studies use different surrogate reference standards, often a combination of imaging modalities and follow-up of lesions.

PET analogues of [^123^I]mIBG, [^18^F]mFBG, and [^124^I]mIBG both visualize NET uptake in neuroblastoma. [^124^I]mIBG is less appealing for diagnostic imaging because of its higher radiation exposure, 2-days imaging, and need for thyroid protection, but may have a purpose for dosimetry before [^131^I]mIBG therapy. Given the potentially high diagnostic accuracy of [^18^F]mFBG PET-CT, it is possible to further reduce radiation exposure [52]. [^18^F]F-DOPA, [^68^Ga]Ga-DOTA peptides, and [^18^F]FDG use molecular targets other than NET and are increasingly used in mIBG non-avid neuroblastoma. Currently, [^18^F]FDG is the only PET-tracer that has been recommended by the guidelines as alternative in the evaluation of mIBG-negative neuroblastoma or discrepancy between mIBG imaging and radiological/clinical findings [18]. The major limitation of [^18^F]FDG is the physiological brain and variable bone marrow activity (f.e. reactive bone marrow activity after therapy) and, as a consequence, the impaired detection of skull and bone marrow lesions. In this case, [^18^F]F-DOPA and [^68^Ga]Ga-DOTA peptides may be more useful, especially when skull lesions are suspected. The advantages of [^68^Ga]Ga-DOTA peptide imaging are high focal tumor-to-background contrast (high SUV values) even for small lesions, a lower radiation exposure, no requirement of a cyclotron, and the possibility of PRRT. However, [^68^Ga]Ga-DOTA peptide uptake is not tumor-specific and can likewise be present in benign pathology. The prognostic value of quantified disease burden on PET has been demonstrated for [^18^F]FDG and [^18^F]F-DOPA. Measurements of tracer uptake in tumor lesions could identify patients who are more likely to relapse and are associated with poor outcome [74,107,108].

Other than the limited availability, the most important limitation that prevents the use of these new PET tracers is the limited experience and lack of knowledge, for example, on the normal distribution and radiation burden in young children. This may be overcome in the future if more centers install cyclotrons and/or generators and more studies are conducted. Currently, there are multiple ongoing prospective clinical trials comparing these PET tracers to [^123^I]mIBG in patients with neuroblastoma, for instance, trial NL8152 and NCT02348749 on [^18^F]mFBG and NCT02043899 on [^124^I]mIBG. As suggested by Piccardo and colleagues, comparison of [^18^F]F-DOPA or [^68^Ga]Ga-DOTA peptides should be conducted with a PET analogue of [^123^I]mIBG to eliminate any bias related to different imaging techniques [107].

The difficulty of introducing a more sensitive PET tracer is the uncertainty of the clinical relevance when more lesions are detected because this can have important implications on the overall management of patients. Improved imaging accuracy will likely lead to the upstaging of some patients. Nevertheless, a more accurate determination of disease extent could provide critical information and may lead to adequate intensification of treatment in children with inferior outcomes. Around 50–60% of patients deemed to be in complete remission on [^123^I]mIBG imaging at end of treatment eventually relapse [8]. We hypothesize that persisting oligometastases only detectable by PET imaging, implying that these patients were not in complete remission, may be the cause of recurrent disease. In this case, these patients could benefit from maximum cytoreductive treatments, for instance, additional targeted radio(nuclide) therapy.

Before the current standard [^123^I]mIBG imaging can be replaced by PET imaging, re-evaluation of diagnostic and treatment strategies is warranted. The prognostic value of current semiquantitative scoring systems is only validated for disease burden on mIBG scintigraphy. New scoring systems, ideally implementing quantification of tracer uptake, should be validated for these PET tracers.

It is unclear in which setting a patient may benefit most from imaging with a certain tracer. [^18^F]DOPA could be useful in neuroblastoma with insufficient NET or SSTR expression, but this has not been studied yet. Variable tracer uptake on an interpatient, intrapatient and intratumoral level once again illustrates the heterogenous character of neuroblastoma and is a great challenge in the clinical practice. The exact reason for reduced NET or SSTR expression or a modified uptake mechanism is not fully understood but may partially be linked to tumor dedifferentiation. Heterogeneity of neuroblastoma indicate that that imaging with one molecular target may not fully depict disease extent. Therefore, expanding to imaging with a combination of radiotracers that use different molecular targets should be considered. [^18^F]FDG PET-CT has already proven to complement [^123^I]mIBG in selected patients with [^123^I]mIBG-negative tumors. Despite extra radiation exposure and costs, routine implementation of [^18^F]FDG PET-CT may have a role in high-risk patients to increase diagnostic accuracy while providing prognostic information. Furthermore, routine immunohistochemical identification of (molecular) target expression in all tumor samples may guide nuclear imaging and therapy. Molecular target heterogeneity needs to be studied in prospective studies and correlated to activity on PET imaging and clinical outcomes.

With ongoing developments of PET technologies, further improvement of image quality and reduction in radiation exposure can be expected. In the future, hybrid PET-MRI could eliminate the radiation exposure of CT and provide high soft tissue contrast images. In addition, improved tumor delineation may provide important information for radiotherapy planning. All in all, PET is expected to play an important role in the future of neuroblastoma imaging.

## 10. Conclusions

The advantages of PET imaging and the preliminary results on the diagnostic accuracy of new PET tracers for lesion detection in neuroblastoma, compared to current standard [^123^I]mIBG imaging, are promising. PET imaging could have an important role in demonstrating full disease extent and guiding adequate therapy with the ultimate aim of improving outcome in patients at high risk for refractory and recurrent neuroblastoma. Heterogenous tracer uptake in tumor lesions can be a challenge in the management of neuroblastoma, with the risk of missing lesions. Therefore, it is important to investigate various tracers with different uptake mechanisms and possibly expand to multimodality nuclear imaging. Larger multicenter investigations are needed in order to establish the role of these new tracers in the management of neuroblastoma patients.

## Appendix A

**Table A1:** characteristics of radiotracers for nuclear imaging of neuroblastoma.

Tracer	Molecular Target	Radioisotope (Half-Life)	Advantages	Limitations
[^123^I]mIBG	norepinephrine transporter	iodine-123 (13 h)	- validated scoring systems - possible to select patients for [^131^I]mIBG therapy	- two-day imaging - scintigraphy/SPECT imaging (limited spatial resolution, limited tomographic range, long acquisitions, as opposed to PET imaging) - need for thyroid protection - negative in 10% neuroblastomas
[^124^I]mIBG	norepinephrine transporter	iodine-124 (100 h)	- PET imaging - possibility for [^131^I]mIBG therapy dosimetry	- two-day imaging - high radiation exposure - poor image quality (only 23% positron abundance) - need for thyroid protection - limited experience & availability
[^18^F]mFBG	norepinephrine transporter	fluorine-18 (110 min)	- PET imaging - one-day imaging	- limited experience & availability
[^18^F]FDG	glucose metabolism	fluorine-18 (110 min)	- PET imaging - one-day imaging - widely available - often positive in mIBG non-avid neuroblastoma - prognostic value	- not tumor-specific - impaired detection of bone marrow and skull lesions - negative in neuroblastomas with low glucose metabolism - fasting - not possible to select patients for [^131^I]mIBG therapy
[^18^F]F-DOPA	catecholamine metabolism	fluorine-18 (110 min)	- PET imaging - one-day imaging - prognostic value	- fasting - not possible to select patients for [^131^I]mIBG therapy - limited experience & availability
[^68^Ga]Ga-DOTA peptides	somatostatin receptors	gallium-68 (68 min)	- PET imaging - one-day imaging - relatively low radiation exposure - gallium generator (often easier available than cyclotrons) - possible to select patients for peptide receptor radionuclide therapy	- not specific for tumors of sympathetic nervous system - negative in neuroblastomas with low SSTR expression - limited experience
[^11^C]mHED	norepinephrine transporter	carbon-11 (20 min)	- PET imaging - one-day imaging - relatively low radiation exposure	- rigid time schedule - limited experience & availability

Abbreviations: *mIBG meta*-iodobenzylguanidine, *mFBG meta*-fluorobenzylguanidine, *FDG* fluorodeoxyglucose, *F-DOPA* fluoro-L-dihydroxyphenylalanine, *mHED meta*-hydroxyephedrine, *SPECT* single-photon emission computed tomography *PET* positron emission tomography.
